# Endovascular management of a large hepatic artery aneurysm related to type B aortic dissection

**DOI:** 10.1259/bjrcr.20200009

**Published:** 2020-08-05

**Authors:** John Reicher, Demetris Tsiakkis, Barnabas R Green, Paul Walker

**Affiliations:** 1 South Tees Hospitals NHS Foundation Trust, Middlesbrough, UK

## Abstract

Management of visceral artery aneurysms can be challenging: there is limited evidence to determine size thresholds for intervention and it is often technically difficult to exclude the aneurysms while preserving visceral perfusion.

We present the case of a 68-year-old male with a rapidly enlarging hepatic artery aneurysm related to type B aortic dissection extending into the coeliac axis, which presented unique difficulties due to its morphology and filling via the false lumen. Endovascular treatment involved stent–graft placement from the coeliac axis into the splenic artery with the intention of excluding the coeliac supply to the common hepatic artery. Despite early stent–graft occlusion, the aneurysm was successfully excluded and adequate hepatic and splenic perfusion was maintained. The patient made a good recovery.

## Introduction

True visceral artery aneurysms (VAAs) are rare, with an estimated incidence of 0.1–0.01%. The splenic and hepatic arteries are the most commonly affected vessels. Causative factors include atherosclerosis, pregnancy, infection, and connective tissue disorders.^1^ Multiple strategies for endovascular treatment have been described, which balance the need to exclude the aneurysm while also preserving visceral perfusion.^2,3^ We present a case of hepatic artery aneurysm with an unusual aetiology and specific anatomical challenges which was managed endovascularly with a good clinical outcome.

## Clinical presentation

A 68-year-old male presented to his General Practitioner (GP) with severe, transient abdominal and back pain. He had a history of radical prostatectomy, hypertension, and gout. His regular medications were amlodipine, simvastatin, and allopurinol.

Initial blood tests showed mild normocytic anaemia (haemoglobin 126 g l^−1^), normal renal function, and deranged liver enzymes (ALT 247 U l^−1^, ALP 441 U l^−1^) with normal bilirubin (8 micromol/l). Abdominal ultrasound was reported as showing a distended gallbladder with echogenic contents, but no other significant finding. Thoracolumbar spine MRI showed no spinal pathology but a dissection flap was noted in the aorta.

On review in Vascular Surgery outpatient clinic 2 weeks after symptom onset, clinical examination revealed a blood pressure of 152/98 mmHg in the left arm, 165/98 in the right arm, a soft and non-tender abdomen, and normal lower limb pulses bilaterally.

## Imaging findings

**Figure 1.**
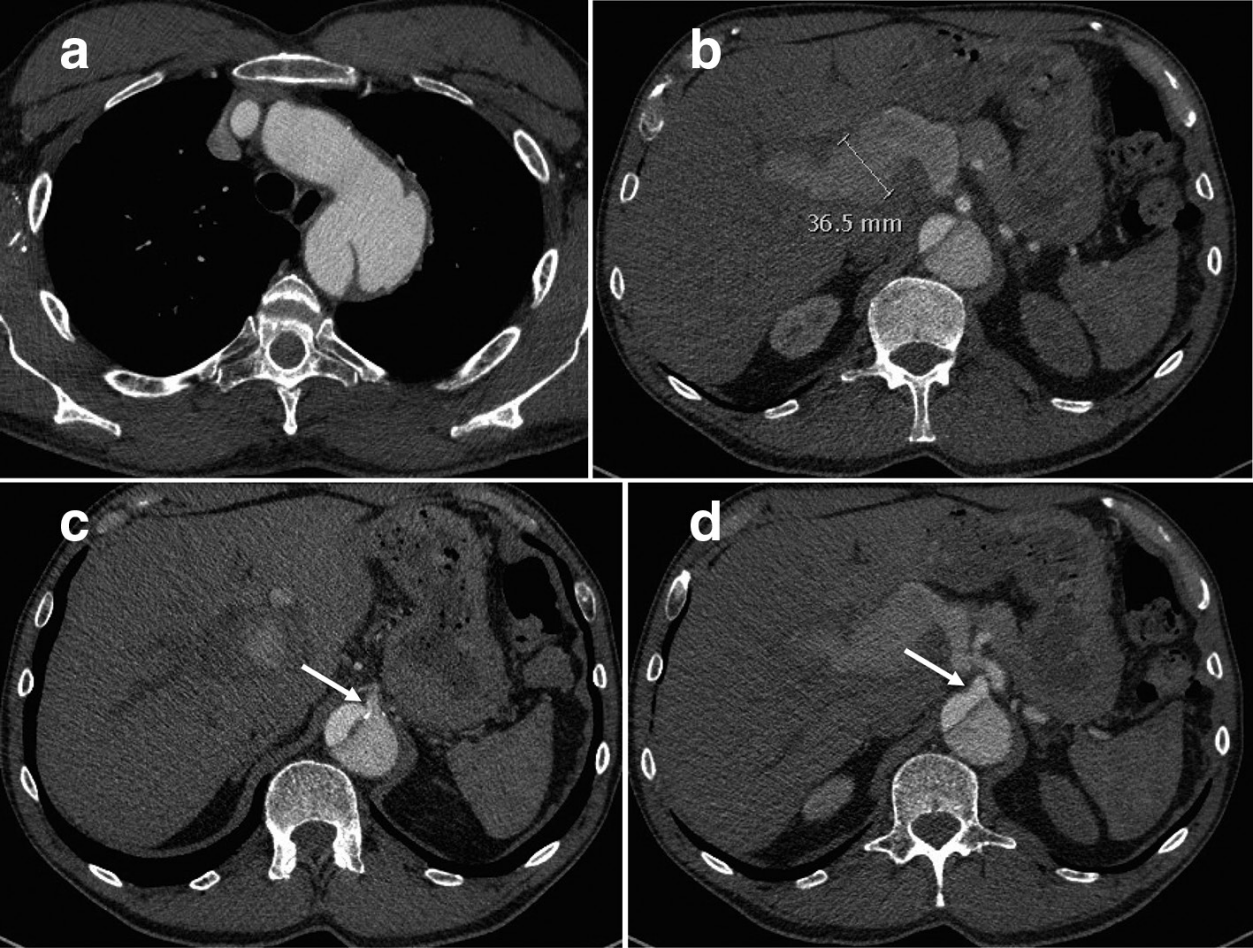
Axial slices from the initial scan – an arterial phase examination performed on a 128-slice Somatom Definition AS + CT scanner (Siemens, Germany) with 90 ml of Omnipaque 300 contrast medium (GE, USA) using bolus-tracking technique; reconstructed into 3 mm slices. All subsequent included scans insubsequent figures were performed with the same parameters unless otherwise indicated. (a) Thoracic slice showing Type B aortic dissection with a wide intimal defect 3 cm distal to the left subclavian artery origin; (b) upper abdominal slice showing a 37 mm fusiform hepatic artery aneurysm, with degree of enhancement similar to the aortic false lumen; (c) shows the aortic intimal flap extending up to the origin of the coeliac artery (arrow - please note this arrow has moved since the figure has been reformatted); (d) shows the SMA origin is supplied by the true lumen (arrow - please note this arrow has moved since the figure has been reformatted). SMA, superior mesenteric artery.

A CT aortogram (arterial phase, Figure 1) showed a Stanford type B aortic dissection with an intimal defect 3 cm distal to the left subclavian artery origin and a dissection flap extending to the aortic bifurcation. The descending thoracic aorta was aneurysmal at 45 mm, but the other aortic segments were non-aneurysmal. The superior mesenteric artery (SMA), right renal artery, and right common iliac artery were supplied by the true lumen; left renal artery was supplied by the false lumen; coeliac axis and left common iliac artery had joint supply from true and false lumens. There was a tubular structure at the porta hepatis measuring up to 37 mm diameter, with similar density to the aortic false lumen. This was thought to correspond to the echogenic structure reported as a distended gallbladder on ultrasound. A hepatic artery aneurysm was suspected.

**Figure 2.**
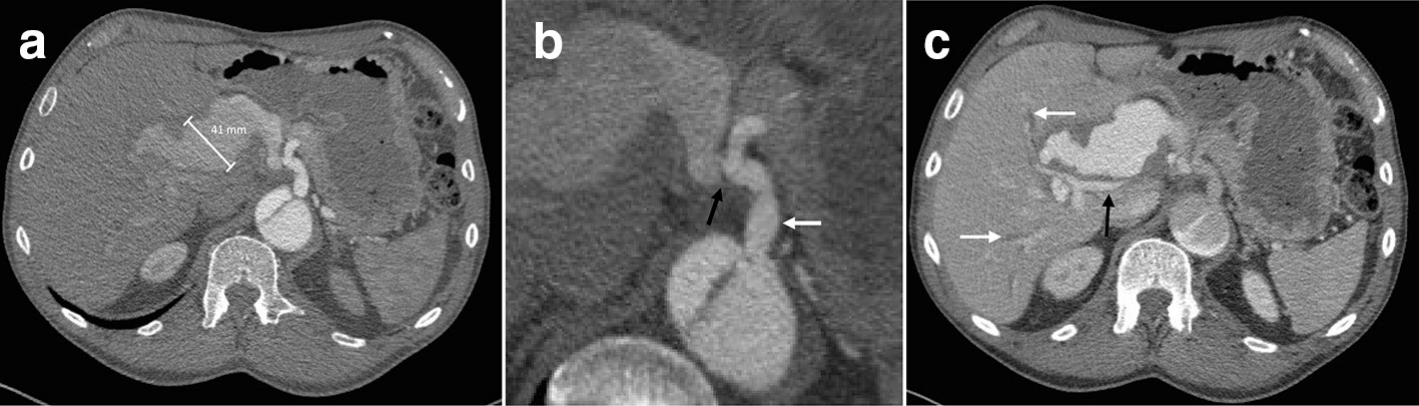
Repeat CT 1 week later, using the same imaging parameters as the previous scan with the addition of a portal venous phase performed 70 s following commencement of contrast injection. (a) The arterial phase study shows interval enlargement of the aneurysm to 41 mm; (b) zoomed axial image demonstrating the course of the coeliac artery (white arrow- please note this arrow has moved since the figure has been reformatted), with an intimal flap across the common hepatic artery origin (black arrow- please note this arrow has moved since the figure has been reformatted). (c) The portal venous phase study shows compression of the portal vein (black arrow) and intrahepatic biliary dilatation (white arrows).

The CT was repeated 1 week later (Figure 2), this time with arterial and portal venous phases. This confirmed a fusiform aneurysm involving the common hepatic, proper hepatic, and left and right hepatic arteries. This had grown to 41 mm in maximal diameter. An intimal flap was demonstrated at the origin of the common hepatic artery, suggesting aneurysm perfusion via the false lumen. The aneurysm was compressing the extrahepatic bile ducts, with mild upstream intrahepatic biliary dilatation, and the main portal vein. The gastroduodenal artery (GDA) was not clearly demonstrated, possibly reflecting compression or effacement by the aneurysm.

Repeat blood tests showed worsening liver enzymes (ALT 324 U l^−1^, ALP 1125 U l^−1^) and newly elevated bilirubin (152 micromol/l).

## Treatment

**Figure 3.**
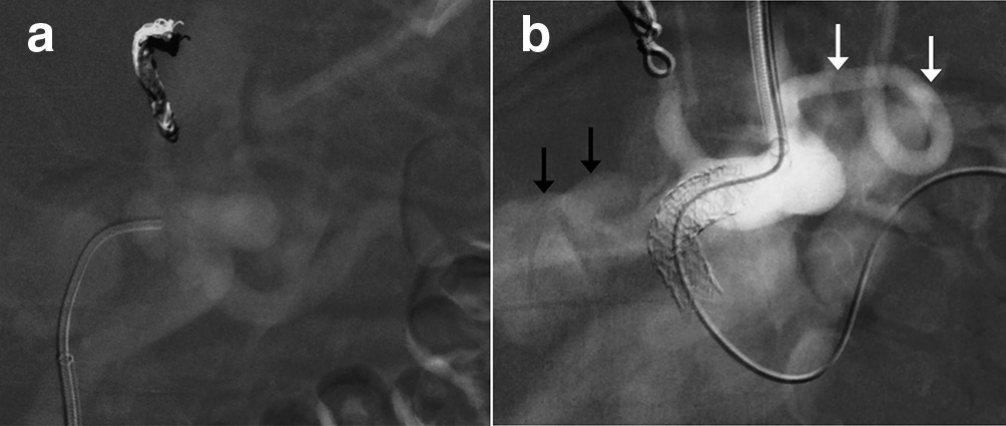
Intraoperative imaging. (a) Pre-stenting coeliac angiogram shows successful coil embolisation of the left gastric artery; (b) Post-stenting coeliac angiogram shows adequate placement of the stent–graft across the origin of the common hepatic artery. At this phase, there is no filling of the splenic artery, suggesting poor flow through the stent–graft. There is some residual filling of the aneurysm (black arrows), but this was no longer demonstrated after dilatation of the segment of stent–graft within the coeliac axis to 9 mm. The white arrows show the left inferior phrenic artery, arising immediately adjacent to the coeliac origin.

After multidisciplinary discussion involving specialists in interventional radiology, hepatobiliary surgery and vascular surgery, it was determined that urgent intervention was warranted due to the rapid enlargement of the aneurysm (4 mm in 7 days) and the deteriorating blood tests. It was decided to place a stent–graft from the coeliac axis into the splenic artery, with the intention of excluding coeliac supply to the common hepatic artery while maintaining splenic perfusion.(Figure 3)

Via right common femoral artery access under local anaesthetic, the left gastric artery was embolised with coils to reduce the risk of endoleak. Stable access to the splenic artery could not be achieved via the femoral sheath; therefore left brachial artery access was gained and a long 6-French sheath was advanced into the proximal splenic artery. A 7 × 37 mm balloon-mounted stent–graft (BeGraft, Bentley, Germany) was deployed from the coeliac artery to the proximal splenic artery, covering the common hepatic artery origin. The segment of stent-graft within the coeliac artery was over dilated with a 9 × 20 mm balloon. A final angiogram confirmed occlusion of the hepatic artery origin, with no filling of the aneurysm from the coeliac artery. Flow into the splenic artery through the stent was sluggish – this was thought to be due to splenic artery spasm downstream of the stent–graft. The original procedural plan was to perform angiography of the GDA via the pancreaticoduodenal arcade from the SMA to assess for retrograde aneurysm perfusion following stent–graft deployment and collateral supply to the liver. However, the patient became restless and it was decided to stop the procedure and reassess the aneurysm and hepatic arterial supply with early post-operative CT instead. It was decided not to institute antiplatelet or anticoagulant therapy following the procedure, as the risk of haemorrhage from the aneurysm was thought to outweigh any benefits of optimising stent–graft patency.

**Figure 4.**
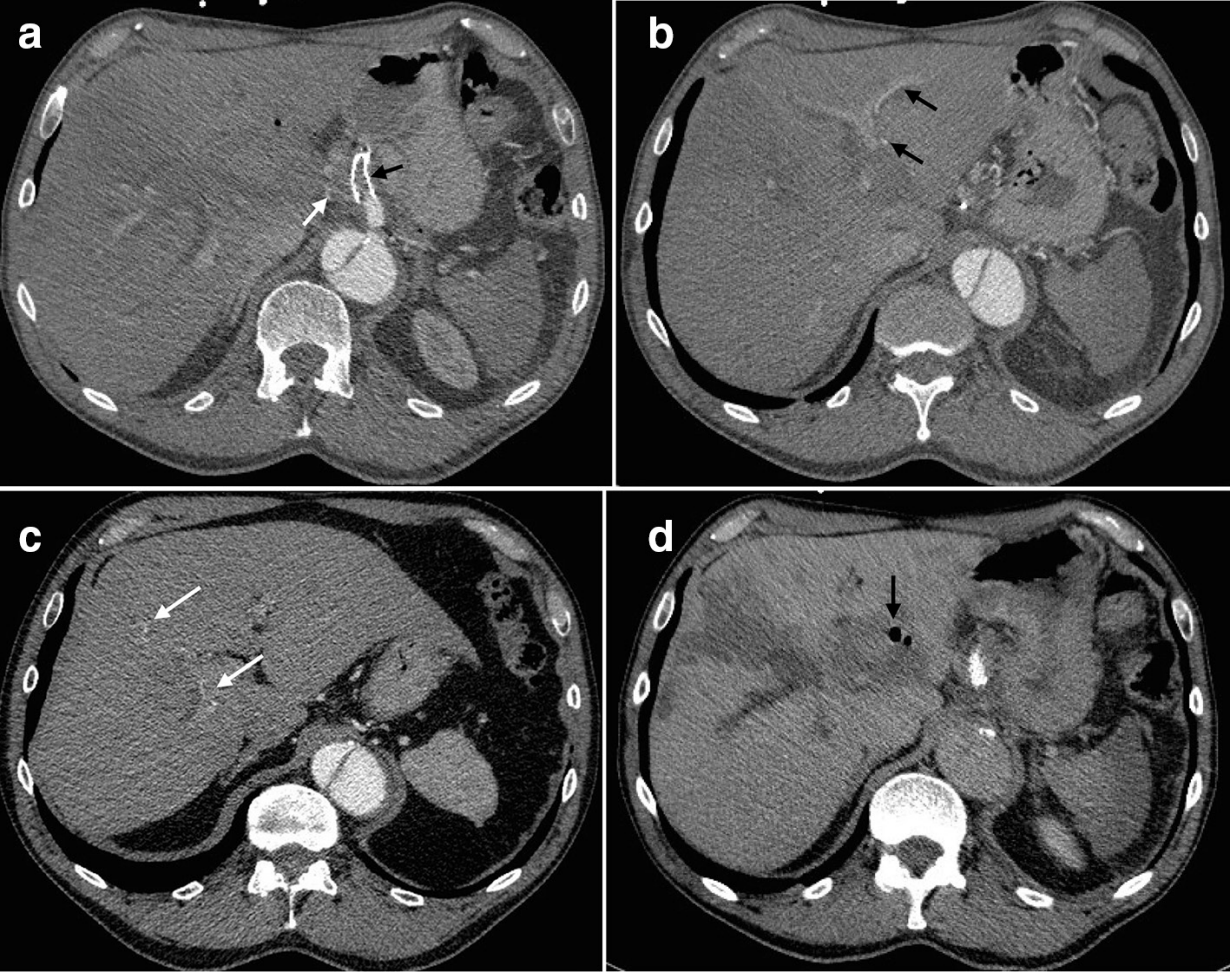
CT performed 3 days post-operatively. (a) Arterial phase imaging shows the stent–graft (black arrow) and common hepatic artery (white arrow) are occluded; Intra hepatic arterial branches filling via collaterals in the left lobe (b, black arrows) and right lobe (c, white arrows - please note these white arrows have moved since the figure has been reformatted); (d) Venous phase imaging showing segment IV hepatic infarction, hypoperfusion in the periphery of the spleenand gas in the excluded aneurysm (black arrow).

In the early post-operative period, the patient experienced mild abdominal discomfort and a low-grade fever. CT imaging (Figure 4) on day 3 showed that the stent–graft was occluded, but the majority of the spleen was perfused via collaterals. No contrast filling of the aneurysm was demonstrated, confirming successful exclusion of the common hepatic artery. There was infarction in hepatic segment IV, but multiple arterial branches in both hepatic lobes were filling via collaterals and the majority of the liver appeared to be enhancing normally. After a short course of empirical antibiotics and symptomatic treatment, the patient made a rapid clinical recovery. There was steady improvement in the liver enzymes and bilirubin normalised within 10 days.

**Figure 5.**
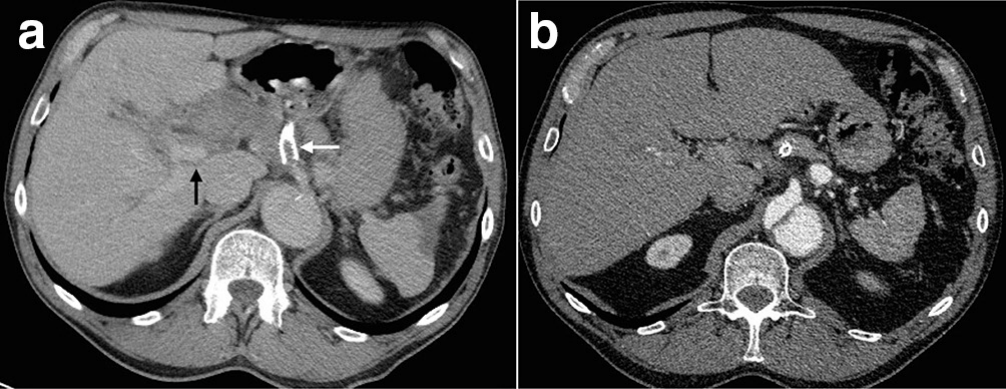
(a) Portal venous phase CT scan performed 1 month post-operatively showing the segment IV hepatic parenchymal changes have improved and the excluded aneurysm has started to reduce in size, with improvement of the portal vein compression (black arrow). The stent–graft remains occluded (white arrow). (b) 2 year follow-up arterial phase CT scan shows involution of the occluded aneurysm.

The patient is well 2 years after the procedure. Follow-up CTs have shown resolution of the hepatic artery aneurysm and no significant long-term complications (Figure 5). The stent-graft remains occluded. The descending thoracic aortic diameter is stable.

## Discussion

While it is generally accepted that all visceral artery *pseudo*-aneurysms require intervention, regardless of size or location, there is some uncertainty around thresholds for intervention for *true* VAAs. The most widely accepted practice is to treat true aneurysms with a diameter of 2 cm or more, in the absence of symptoms.^4,5^ While in certain situations it can be difficult to distinguish between true and pseudo-aneurysms on imaging alone, in our patient, we considered that the imaging findings were more characteristic of a true aneurysm, given that the aneurysm extended beyond the proper hepatic artery bifurcation into the left and right hepatic arteries, and there was no surrounding haematoma. In any case, given the rapid enlargement to 4.1 cm and associated biliary obstruction and portal vein compression, urgent intervention was clearly mandated. Given the increased perioperative mortality risk associated with surgical repair of VAAs, endovascular repair is preferable in the majority of cases.^5,6^ In our patient, surgery was felt to involve unacceptable risks, especially given the anatomy of the aneurysm. The proposed surgical approach under consideration would potentially have required ligation of the common hepatic artery and the left and right hepatic arteries, with a high risk of associated ischaemic complications in addition to the risks of major laparotomy and retroperitoneal dissection.

A variety of different endovascular strategies have been described involving coils, vascular plugs, liquid embolic agents, and stent–grafts. Usually treatment decisions relate to aneurysm anatomy with the requirement to exclude the aneurysm being balanced with the need to maintain visceral perfusion.^1–3,5,7^ In general, assuming a stent–graft cannot be placed through an entire aneurysm, or across the neck of a pseudo-aneurysm, it is recommended in non-terminal vascular territories (such as the hepatic circulation) to occlude both upstream and downstream of the aneurysm to prevent both antegrade and retrograde perfusion.^5,6^ In our patient, it was not possible to place a stent–graft through the whole aneurysm given its length and extension beyond the bifurcation of the proper hepatic artery. The normal rules of upstream and downstream occlusion, which would have mandated the embolisation of the common hepatic artery at its origin as well as the left and right hepatic arteries, did not apply in this case given that the aneurysm was perfused via the false lumen. Our judgement was that occlusion of the common hepatic artery origin alone would be sufficient as there would be a low risk of retrograde perfusion of the false lumen aneurysm via normal collateral pathways (such as the GDA), especially given the SMA origin was supplied exclusively by the true lumen. Furthermore, embolisation of the left and right hepatic arteries downstream of the aneurysm would present an unacceptable risk of hepatic ischaemia, especially given the presence of portal vein compression. Our treatment strategy of stent–graft placement from coeliac axis to splenic artery was intended to prevent antegrade perfusion of the common hepatic artery false lumen (and therefore exclude the aneurysm), while allowing true-lumen collateralisation from the GDA to preserve hepatic perfusion. Thankfully, the strategy was successful: upstream occlusion alone proved to be sufficient to treat the aneurysm while adequate hepatic arterial supply was preserved to prevent clinically significant hepatic ischaemia; and despite early stent–graft occlusion, collateral supply also prevented major splenic infarction.

A final learning point from this case is the need to assess the visceral arteries carefully when reporting CT imaging, especially in the setting of aortic dissection. Although VAA related to aortic dissection is rare, there is a recognised association.^8^ It is important not to miss opportunities to diagnose VAAs, given the potential to intervene prophylactically to prevent aneurysm rupture.

## Learning points

- Although rare, there is an association between aortic dissection and VAA. Therefore it is important to assess carefully the visceral arteries on imaging performed for aortic dissection.
- When technically feasible, endovascular therapy is generally preferred to open surgery for VAA.
- Any intervention must exclude the aneurysm while maintaining adequate perfusion to the major viscera.
- Treatment options include coil embolisation, vascular plugs and stent–graft placement, depending on aneurysm anatomy.

## Normal ranges

Alanine aminotransferase (ALT): 0–40 units/l

Alkaline phosphatase (ALP): 30–120 units/l

Bilirubin: 0–21 micromol/l

Haemoglobin: 135–170 g dl^−1^
